# Androgen receptor expression in male breast carcinoma: lack of clinicopathological association

**DOI:** 10.1038/sj.bjc.6690153

**Published:** 1999-02

**Authors:** A Pich, E Margaria, L Chiusa, G Candelaresi, O Dal Canton

**Affiliations:** Department of Biomedical Sciences and Human Oncology, Section of Pathology, University of Turin, Via Santena 7, Turin, I-10126, Italy; Division of Pathology, S. Giovanni Hospital, Via Cavour 31, Turin, Italy; Division of Oncology, S. Giovanni Hospital, Via Cavour 31, Turin, I-10123, Italy

**Keywords:** androgen receptors, male breast carcinoma, immunohistochemistry, prognosis

## Abstract

Androgen receptor (AR) expression was retrospectively analysed in 47 primary male breast carcinomas (MBCs) using a monoclonal antibody on formalin-fixed, paraffin-embedded tissues. AR immunopositivity was detected in 16 out of 47 (34%) cases. No association was found with patient age, tumour stage, progesterone receptor (PGR) or p53 protein expression. Well-differentiated MBCs tended to be AR positive more often than poorly differentiated ones (*P*= 0.08). A negative association was found between ARs and cell proliferative activity: MIB-1 scores were higher (25.4%) in AR-negative than in AR-positive cases (21.11%; *P*= 0.04). A strong positive association (*P*= 0.0001) was found between ARs and oestrogen receptors (ERs). In univariate analysis, ARs (as well as ERs and PGRs) were not correlated with overall survival; tumour histological grade (*P*= 0.02), size (*P*= 0.01), p53 expression (*P*= 0.0008) and MIB-1 scores (*P*= 0.0003) had strong prognostic value. In multivariate survival analysis, only p53 expression (*P*= 0.002) and histological grade (*P*= 0.02) retained independent prognostic significance. In conclusion, the lack of association between AR and most clinicopathological features and survival, together with the absence of prognostic value for ER/PGR status, suggest that MBCs are biologically different from female breast carcinomas and make it questionable to use antihormonal therapy for patients with MBC. *© 1999 Cancer Research Campaign*


*British Journal of Cancer* (1999) **79**(5/6), 959–964
© 1999 Cancer Research Campaign
Article no. bjoc.1998.0153

### Androgen receptor expression in male breast carcinoma: lack of clinicopathological association

**A Pich[1], E Margaria[2], L Chiusa[1], G Candelaresi[2] and O Dal Canton[3]**

[1]Department of Biomedical Sciences and Human Oncology, Section of Pathology, University of Turin, Via Santena 7, I-10126 Turin, Italy; Divisions of [2]Pathology and [3]Oncology, S. Giovanni Hospital, Via Cavour 31, I-10123 Turin, Italy

**Summary** Androgen receptor (AR) expression was retrospectively analysed in 47 primary male breast carcinomas (MBCs) using a monoclonal antibody on formalin-fixed, paraffin-embedded tissues. AR immunopositivity was detected in 16 out of 47 (34%) cases. No association was found with patient age, tumour stage, progesterone receptor (PGR) or p53 protein expression. Well-differentiated MBCs tended to be AR positive more often than poorly differentiated ones ($P = 0.08$). A negative association was found between ARs and cell proliferative activity: MIB-1 scores were higher (25.4%) in AR-negative than in AR-positive cases (21.11%; $P = 0.04$). A strong positive association ($P = 0.0001$) was found between ARs and oestrogen receptors (ERs). In univariate analysis, ARs (as well as ERs and PGRs) were not correlated with overall survival; tumour histological grade ($P = 0.02$), size ($P = 0.01$), p53 expression ($P = 0.0008$) and MIB-1 scores ($P = 0.0003$) had strong prognostic value. In multivariate survival analysis, only p53 expression ($P = 0.002$) and histological grade ($P = 0.02$) retained independent prognostic significance. In conclusion, the lack of association between AR and most clinicopathological features and survival, together with the absence of prognostic value for ER/PGR status, suggest that MBCs are biologically different from female breast carcinomas and make it questionable to use antihormonal therapy for patients with MBC.

**Keywords:** androgen receptors; male breast carcinoma; immunohistochemistry; prognosis

Male breast carcinoma (MBC) represents only 1% of all mammary cancers (Hecht and Winchester, 1994) and seems to behave more aggressively than female breast carcinomas (FBCs) (Ribeiro, 1985; Salvadori et al, 1994), although no difference in survival between MBC and FBC has been reported (Guinee et al, 1993; Cutuli et al, 1995; Weber-Chappuis et al, 1996). Treatment of MBC is far from standardization because of the uncommon nature of the disease, and no randomized comparisons of treatment have been carried out as in FBC. Modified radical mastectomy and irradiation for cases with risk factors of local relapse and adjuvant tamoxifen have been claimed as the optimal treatment in a large series of patients with MBC (Cutuli et al, 1995).

Hormone receptor characterization of FBC is well established, and the presence of oestrogen and progesterone receptors (ERs, PGRs) suggests a tumour likely to respond to endocrine therapy (Hähnel, 1985). ERs have also been extensively investigated in MBC (Everson et al, 1980; Friedman et al, 1981; Dawson et al, 1992; Fox et al, 1992; Rogers et al, 1993; Pich et al, 1994, 1996; Bruce et al, 1996; Joshi et al, 1996; Weber-Chappuis et al, 1996; Williams et al, 1996; Willsher et al, 1997). The frequency of ER expression is higher in MBC than FBC (Dawson et al, 1992; Weber-Chappuis et al, 1996) and tamoxifen was reported to be a useful adjuvant therapy in patients with advanced MBC (Bezwoda et al, 1987; Ribeiro and Swindell 1992; Cutuli et al, 1995).

Few studies exist on the role of androgen receptors (ARs) in breast cancer. Androgens cause regression of DMBA-induced breast cancers in rats (Teller et al, 1966) and suppress growth in human breast cancer cell lines (Poulin et al, 1988). ARs have been detected in 31–91% FBCs (Allegra et al, 1979; Bryan et al, 1984; Lea et al, 1989; Soreide et al, 1992; Isola, 1993; Kuenen-Boumeester et al, 1996). No association was found with tumour size, lymph node status (Allegra et al, 1979; Miller et al, 1985), or tumour stage (Langer et al, 1990), although high AR levels seem to predict lymph node metastases (Soreide et al, 1992). AR-positive patients respond better to hormone therapy (Nomura et al, 1980; Bryan et al, 1984; Birrell et al, 1995) and have longer disease-free or overall survival rates (Bryan et al, 1984; Langer et al, 1990; Birrell et al, 1995; Kuenen-Boumeester et al, 1996).

In only a few series of MBCs have ARs been investigated (Calandra et al, 1984; Mercer et al, 1984; Pacheco et al, 1986; Sasano et al, 1996). AR positivity ranged from 57% to 87%; a direct relationship between AR and other steroid hormone receptors was reported (Pacheco et al, 1986), although this was not recently confirmed (Sasano et al, 1996). However, the number of the patients is small (5–19), and no correlation with prognosis has been reported so far.

In this work, we have retrospectively investigated the expression of ARs in 47 primary MBCs at diagnosis, using immunohistochemistry on sections from formalin-fixed, paraffin-embedded tissues. The aim was to assess whether ARs were associated with tumour clinicopathological features, ER and PGR, p53 protein expression, cell proliferative activity and patient survival.

*Received 19 March 1998*
*Revised 6 July 1998*
*Accepted 14 July 1998*

*Correspondence to:* A Pich, Dipartimento di Scienze Biomediche e Oncologia Umana, Università di Torino, Via Santena 7, I-10126 Torino, Italy

### MATERIALS AND METHODS

### Patients and tumours

Forty-seven MBCs were collected from the files of the pathology sections of the Department of Biomedical Sciences and Human

**Table 1** AR expression in MBC according to clinicopathological features, ER/PGR status, p53 expression and cell proliferative activity

| Variable | *n* | AR-positive cases (%) | AR-negative cases (%) | *P*-value |
|---|---|---|---|---|
| Whole series | 47 | 16 (34) | 31 (66) | |
| Age (years) | | | | |
| ≤ 45 | 6 | 2 (33.3) | 4 (66.7) | |
| 46–70 | 25 | 9 (36) | 16 (64) | 0.95 |
| > 70 | 16 | 5 (31.3) | 11 (68.7) | |
| Histological grade | | | | |
| G1 | 8 | 3 (37.5) | 5 (62.5) | |
| G2 | 27 | 12 (44.4) | 15 (55.6) | 0.08 |
| G3 | 12 | 1 (8.3) | 11 (91.7) | |
| T-stage | | | | |
| pT1 | 14 | 6 (42.9) | 8 (57.1) | |
| pT2 | 17 | 6 (35.3) | 11 (64.7) | 0.58 |
| pT3 or 4 | 16 | 4 (25) | 12 (75) | |
| N-stage | | | | |
| N0 | 13 | 5 (38.5) | 8 (61.5) | |
| N1–3 | 20 | 7 (35) | 13 (65) | 1 |
| ER (%) | | | | |
| ≤ 10 | 23 | 1 (4.3) | 22 (95.7) | |
| > 10 | 24 | 15 (62.5) | 9 (37.5) | 0.0001 |
| PGR (%) | | | | |
| ≤ 10 | 25 | 7 (28) | 18 (72) | |
| > 10 | 22 | 9 (40.9) | 13 (59.1) | 0.53 |
| p53 immunoreactivity | | | | |
| Negative | 20 | 6 (30) | 14 (70) | |
| Positive | 27 | 10 (37) | 17 (63) | 0.84 |
| MIB-1 scores (mean ± s.d.) | | 21.11 ± 4.7 | 25.4 ± 9.53 | 0.04[a] |

[a]ANOVA.

Oncology of Turin University and S. Giovanni Hospital (Turin, Italy), dating from 1967 to 1991. The mean age of the patients at diagnosis was 61.5 years (27–86). All the patients were treated with surgery: 42 received radical or modified radical mastectomy and five simple mastectomy. Adjuvant post-operative radiation, hormone (tamoxifen) or chemotherapy alone was administered to 12, 4 and 3 patients respectively. Two patients received both radiation and adjuvant hormone therapy, four radiation and chemotherapy, three chemo- and hormone therapy, and four chemo-, hormone and radiation therapy. A minimum follow-up of 4 years for surviving patients or to patient death was available for all the cases. The mean follow-up time was 65 months (range 1–217 months). In each case, multiple samples were fixed in 10% formalin and embedded in paraffin; haematoxylin–eosin, periodic acid–Schiff, and Giemsa-stained sections were used for histology. Carcinomas were classified according to the World Health Organization (Scarff and Torloni, 1968) and pathologically staged according to the International Union Against Cancer (Hermaneck and Sobin, 1992). All were invasive ductal carcinomas; 14 were stage pT1, 17 pT2 and 16 pT3 to 4; lymph node status was available in 33 cases: 13 were N0 and 20 N1–3. Histological grade was assessed according to Elston and Ellis (1991): eight tumours were grade 1, 27 grade 2, and 12 grade 3.

### Androgen receptor staining and scoring

Sections (4 µm thick) on orthoaminosilane-coated slides (Vectabond, Vector Laboratories, Burlingame, CA, USA), cut on the day of immunostaining (freshly cut sections), were dewaxed, rehydrated and brought to water. Endogenous peroxidase activity was blocked by incubation for 5 min in 3% hydrogen peroxide. Slides were placed in a glass box filled with 10 mmol $l^{-1}$ citrate buffer (pH 6.0) and subjected to microwave irradiation at 750 W for three periods of 5 min each, with replacement of evaporated buffer between periods of heating. The sections were then stained with anti-AR monoclonal antibody (mAb) (clone 2F12) (Novocastra, Newcastle, UK) at 1:10 dilution in 0.05% Tween 20 (Sigma, St Louis, MO, USA) in phosphate-buffered saline (PBS), for 18 h at 4°C in a humidified atmosphere. A standard labelled streptavidin biotin (LSAB) technique (Dakopatts, Glostrup, Denmark) was used for visualization with diaminobenzidine as chromogen. Slides were lightly counterstained with haematoxylin and mounted in resin. Normal mouse serum was substituted for primary antibody as a negative control. Freshly cut sections from paraffin-embedded blocks of human hyperplastic prostate were used as a positive control in each staining run. Scoring of AR immunostaining was independently performed by two pathologists (EM and AP), who had no knowledge of ER, PGR or other clinicopathological data, using a standard light microscope equipped with an ocular reticule (original magnification × 15) and a × 40 objective. In each case, 1000 tumour cells were counted from ten randomly selected areas, ensuring that the whole section was scanned. All the reactive nuclei were considered positive, regardless of the staining intensity, and the fraction of positive cells was determined. The interobserver variation was less than 10%. A case was considered positive if more than 10% nuclei

 © Cancer Research Campaign 1999

**Table 2** Correlation between clinicopathological features, hormone receptors, p53 expression and cell proliferative activity with survival in MBC

| Variable | *n* | Median (months) | 5-year survival rate (%) | 10-year survival rate (%) | *P*-value |
|---|---|---|---|---|---|
| Whole series | 47 | 60 | 50 | 18 | |
| Age (years) | | | | | |
| ≤ 45 | 6 | 22 | 33 | 0 | |
| 46–70 | 25 | 77 | 64 | 27 | 0.12 |
| > 70 | 16 | 52 | 35 | 14 | |
| Histological grade | | | | | |
| G1 | 8 | 99 | 87 | 29 | |
| G2 | 27 | 57 | 51 | 26 | 0.02 |
| G3 | 12 | 25 | 25 | 0 | |
| T-stage | | | | | |
| pT1 | 14 | 96 | 85 | 26 | |
| pT2 | 17 | 38 | 34 | 21 | 0.01 |
| pT3 or 4 | 16 | 25 | 37 | 16 | |
| N-stage | | | | | |
| N0 | 13 | 77 | 62 | 26 | |
| N1–3 | 20 | 57 | 45 | 14 | 0.41 |
| AR (%) | | | | | |
| ≤ 10 | 31 | 52 | 44 | 18 | |
| > 10 | 16 | 62 | 62 | 22 | 0.44 |
| ER (%) | | | | | |
| ≤ 10 | 23 | 60 | 52 | 18 | |
| > 10 | 24 | 55 | 49 | 20 | 0.75 |
| PGR (%) | | | | | |
| ≤ 10 | 25 | 73 | 59 | 21 | |
| > 10 | 22 | 52 | 41 | 16 | 0.76 |
| p53 immunoreactivity | | | | | |
| Negative | 20 | 99 | 79 | 27 | |
| Positive | 27 | 33 | 29 | 11 | 0.0008 |
| MIB-1 scores | | | | | |
| ≤ 24 | 25 | 85 | 71 | 24 | |
| > 24 | 22 | 26 | 27 | 11 | 0.0003 |

were stained, according to the criterion most currently applied for assessing ER or PGR immunopositivity on FBC sections.

### ER, PGR, MIB-1 and p53 staining and scoring

Sections (4 μm thick) on poly-L-lysine-coated slides were stained with specific mAbs using the LSAB method (Dakopatts) and diaminobenzidine as chromogen. ER-ICA and PGR-ICA (Abbott Laboratories, North Chicago, IL, USA) were used at kit dilution, following the procedure of Hiort et al (1988). Normal mouse serum was substituted for primary antibody as a negative control. Sections from known ER-/PGR-positive FBCs were used as positive controls. For MIB-1 and p53 staining, sections were microwave pretreated for two periods of 5 min each in a glass box filled with 10 mmol $l^{-1}$ citrate buffer (pH 6.0) at 750 W. p53-specific mAb DO7 (Oncogene Science, Uniondale, NY, USA), at 1:75 dilution and MIB-1 mAb (Immunotech, Marseille, France) at 1:100 dilution were then applied for 2 h at room temperature in a humidified chamber. Sections of cases known to express p53 protein (i.e. high-grade FBCs) or MIB-1 (i.e. poorly differentiated bladder carcinoma) were included in each staining run as positive controls. All sections were independently scored by two pathologists (EM and AP for ER/PGR; LC and AP for MIB-1 and p53) following the same procedure reported for AR staining.

### Statistical analysis

Associations between AR positivity/negativity and clinicopathological tumour features, ER/PGR status, and p53 expression were assessed by the Yates-corrected chi-squared test. Association between AR positivity/negativity and MIB-1 scores, considered as a continuous variable, was evaluated by one-way analysis of variance (ANOVA). Correlation between AR and ER was estimated by Pearson's correlation coefficient, when AR and ER scores were considered as continuous variables. Univariate survival analysis were based on Kaplan–Meier product-limit estimates of survival distribution (Kaplan and Meier, 1958), and differences between survival curves were tested using the generalized Wilcoxon test. The relative importance of all the variables considered in the univariate analysis was estimated using the Cox proportional hazards regression model (Cox, 1972). All data were processed with BioMeDical computer Programs (BMDP) statistical software (programs 6D, 7D, 4F, 1L, 2L) (Dixon et al, 1990).

### RESULTS

### Distribution of AR immunoreactivity in MBCs

AR staining was exclusively nuclear with some variation in intensity from cell to cell. Reaction intensity was generally weak, especially in very old archival material, and weaker than that observed

© Cancer Research Campaign 1999

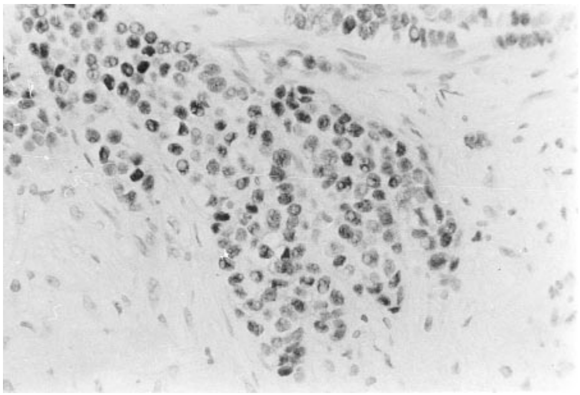

**Figure 1** Immunocytochemical detection of AR in MBC. The intensity of the reaction is rather weak and shows some variation from nucleus to nucleus (AR immunoperoxidase, haematoxylin counterstain, magnification × 260)

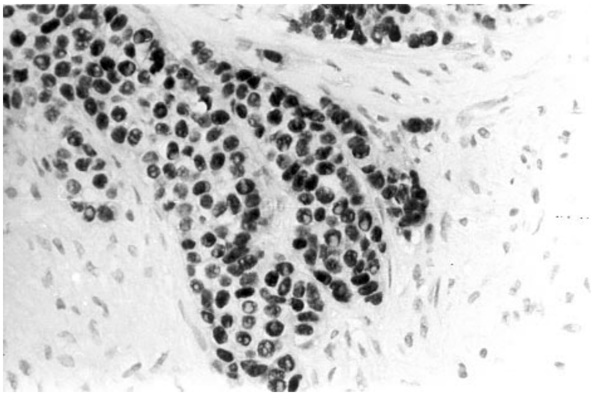

**Figure 2** Immunocytochemical detection of ER in a consecutive serial section of the case illustrated in Figure 1. Most neoplastic cells react more intensely and uniformly than observed for AR staining (ER-ICA immunoperoxidase, haematoxylin counterstain, magnification × 260)

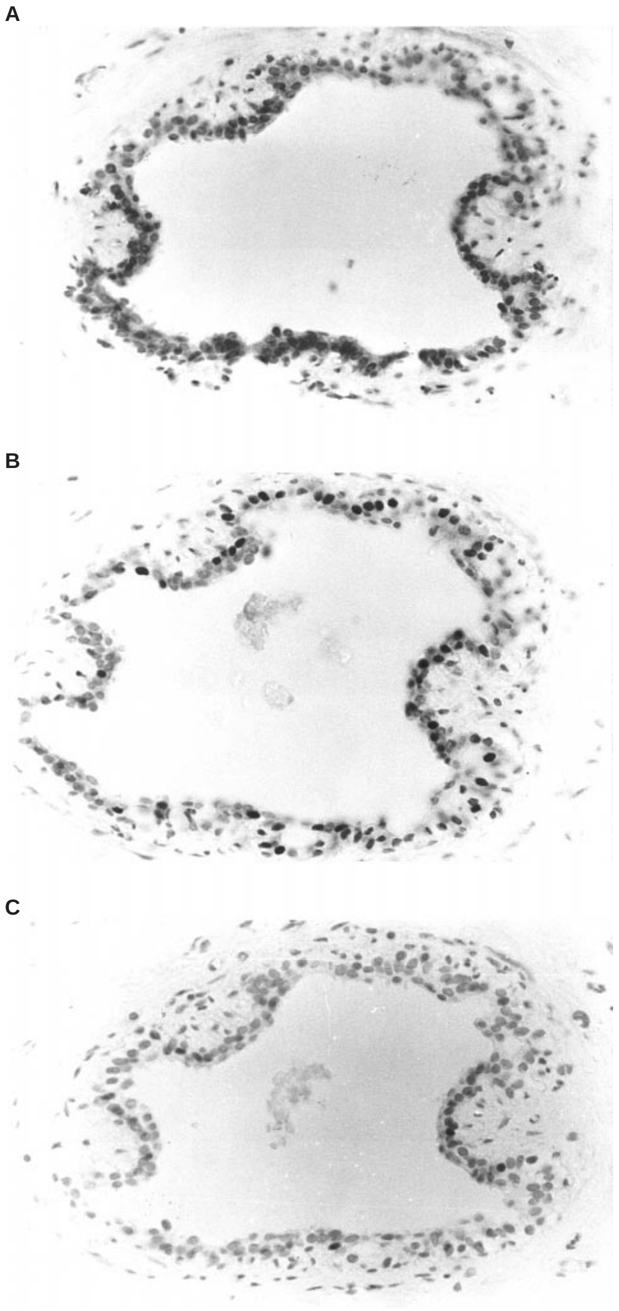

**Figure 3** Immunocytochemical detection of AR (**A**), ER (**B**) and PGR (**C**) in consecutive serial sections of a normal duct of male breast. A weak AR reaction is present in almost all epithelial cells; a stronger ER reaction is seen in many epithelial cells; PGR immunoreactivity is present in only a few nuclei (LSAB, ER-ICA and PGR-ICA immunoperoxidase, haematoxylin counterstain, magnification × 260)

for ER/PGR staining in the same cases (Figures 1 and 2). In normal ductal epithelium, weak AR immunopositivity was present in almost all cells, stronger ER immunopositivity was evident in many cells, and PGR immunostaining was present in only a few cells (Figure 3 A–C). The percentage of AR-positive carcinoma cells varied from 0% to 49.2% (mean 10.64%, s.d. ± 14.93%). Sixteen tumours (34%) had more than 10% stained nuclei (positive cases); 31 (66%) had less than 10% stained nuclei (negative cases). The mean percentage of AR-positive cells for the 16 cases that were deemed positive was 29.24% (s.d. ± 10.73%; range 13.4–49.2%). Only 1 out of 16 cases (6.2%) had a percentage of AR-positive cells (13.4%) near the cut-off value. Among the 14 cases collected up to January 1977, three (21.4%) were AR positive, whereas among the remaining 33 cases 13 (39.4%) were AR positive. However, the difference between the old archived samples and the more recent ones was not significant ($P = 0.39$).

### Association of AR with clinicopathological features, ER/PGR status, p53 and MIB-1 immunoreactivity

No association was found between AR immunopositivity and patient age, tumour stage, PGR and p53 overexpression. A trend towards association was found for histological grade: 37.5% grade 1 and 44.4% grade 2 carcinomas were AR positive, whereas only 8.3% grade 3 were positive ($P = 0.08$). A strong association was seen between AR and ER status: 15 out of 16 AR-positive cases were also ER positive, and 22 out of 31 AR-negative cases were also ER negative ($P = 0.0001$). AR and ER were linearly correlated ($r = 0.51$, $P < 0.001$). A weak negative association was detected between AR and cell proliferative activity: the mean percentage of MIB-1 positive cells was 25.4% in AR negative compared with 21.11% in AR-positive cases ($P = 0.04$). The results are listed in Table 1.

© Cancer Research Campaign 1999

### Univariate survival analysis

At the time of analysis, 36 patients (76.6%) had died of the disease and 11 (23.4%) were alive. The mean follow-up for surviving patients was 114 months (median 102, range 48–217). The median survival for the whole series was 60 months (1–217). The overall 5- and 10-year survival rates were 50% and 18% respectively. AR expression was not associated with survival: the median survival was 52 months for AR-negative compared with 62 months for AR-positive cases ($P = 0.44$). Also, no association was found for ER, PGR and lymph node status. A trend for significance was found for age: patients younger than 45 years or older than 70 had a shorter survival than middle-aged men ($P = 0.12$). Histological grade ($P = 0.02$), tumour size ($P = 0.01$), MIB-1 scores ($P= 0.0003$) and p53 immunoreactivity ($P = 0.0008$) had strong prognostic value. The results are listed in Table 2.

### Multivariate survival analysis

Multivariate survival analysis, performed by testing the association of all variables considered in the univariate analysis in the Cox model, showed that only p53 immunoreactivity ($\chi^2 = 9.59$; $P = 0.002$; hazard ratio 2.71) and histological grade ($\chi^2 = 5.23$; p = 0.02; hazard ratio 1.86) retained independent prognostic significance.

### DISCUSSION

Androgen receptors were detected in 34% MBCs; this rate is far lower than that (57–87%) reported in smaller series of MBCs by other investigators (Calandra et al, 1984; Mercer et al, 1984; Pacheco et al, 1986; Sasano et al, 1996). The discrepancy may be due in part to the different methodologies used for AR demonstration; indeed, most investigators (Calandra et al, 1984; Mercer et al, 1984; Pacheco et al, 1986) performed AR assay from cytosol preparations, which cannot discriminate between receptor-containing malignant and non-malignant cells, or used polyclonal antibodies to detect AR both in neoplastic and stromal cells (Sasano et al, 1996). We performed immunohistochemistry on formalin-fixed, paraffin-embedded tissues, using a monoclonal antibody; with the same procedure, Isola (1993) could not demonstrate AR in routinely fixed material despite testing several antigen unmasking techiques; furthermore, we evaluated AR immunopositivity only in neoplastic cells.

ER and PGR can be reliably assessed on formalin-fixed, paraffin-embedded tissues, with results comparable to those obtained using cytosol assays or frozen-sections (Wilbur et al, 1992; Pertschuk et al, 1996); however, diminished immunoreactivity over time in paraffin-embedded sections stored on glass slides at room temperature has recently been observed during evaluation of several antigens in breast carcinoma (Prioleau and Schnitt, 1995). A significant loss of staining intensity for ER protein occurred on slides stored at room temperature for 12 weeks (Jacobs et al, 1996). In our study, immunohistochemistry for AR was performed the same day of cutting, and we believe that this technique reveals the actual AR status of MBCs. The reliability of the method is also supported by the association between AR and ER (chi-squared 15.19, $P = 0.0001$) or by the linear relationship between AR and ER scores ($r$ 0.51, $P < 0.001$) found in the present series. These results are in accordance with most studies showing association between AR and ER in FBCs (Allegra et al, 1979; Miller et al, 1985; Soreide et al, 1992; Isola, 1993) or MBCs (Pacheco et al, 1986).

We found no significant association between AR and age, in line with a few reports on FBC (Allegra et al, 1979; Bryan et al, 1984; Miller et al, 1985), nor did we find any association with tumour size or lymph node status, in accordance with similar results on FBCs (Allegra et al, 1979; Miller et al, 1985; Langer et al, 1990). A trend towards association ($P = 0.08$) was observed for histological grade: 42.8% G1 or 2 cases, but only 8.3% G3 were AR positive, in agreement with studies on FBCs showing that hormone receptors are expressed at higher rates in well-differentiated tumours (Millis, 1980; Walker et al, 1988).

A weak but significant association could be seen between AR and cell proliferative activity; indeed, AR-negative cases had higher MIB-1 scores (25.4%) than AR-positive cases (21.11%; $P = 0.04$), in accordance with Isola (1993) who showed that FBCs with low S-phase fraction were more often AR positive than rapidly proliferating ones. Our findings are also in agreement with the well-known negative association between ER/PGR and cell proliferation reported in most FBCs (Bertuzzi et al, 1981; Gerdes et al, 1987). No correlation was found between AR expression and patient survival. However, the role of ARs as prognostic factors is still controversial in FBC: AR-positive cases have been associated with better prognosis in some series (Bryan et al, 1984; Langer et al, 1990; Birrell et al, 1995), but AR status did not provide significant prognostic information on relapse-free survival in another large series (Soreide et al, 1992) and did not appear to be an independent variable in multivariate analysis (Kuenen-Boumeester et al, 1996).

Finally, we did not find any prognostic significance for ER/PGR status. This confirms our previous reports in smaller series of MBCs (Pich et al 1994, 1996), but contrasts with the well-known prognostic value of ER/PGR detection in FBC (Alanko et al, 1985; McGuire and Clark, 1985; Pertschuck et al, 1990). We are aware that the number of cases in the present series is relatively small; a larger series would probably have enough power to reveal other weak prognostic factors.

Recently, it was shown that ER and PGR are more expressed in MBC than FBC (Dawson et al, 1992; Weber-Chappuis et al, 1996), but the proteins under oestrogen control (such as pS2, heat shock protein 27 and cathepsin D) are more frequent in FBC than MBC suggesting that ERs in MBC do not have the same function as in FBC (Weber-Chappuis et al, 1996). This could also explain the variable success reported for antihormonal treatment of MBC (Everson et al, 1980; Bezwoda et al, 1987).

In conclusion, lack of association between AR and most clinico-pathological tumour features, and lack of correlation between AR, ER and PGR status with patient survival found in the present series, together with the side-effects of tamoxifen administration reported in MBC (Anelli et al, 1994), make questionable the use of antihormonal therapy for patients with MBC.

### ACKNOWLEDGEMENT

This work was supported by grants from the Italian Ministero dell'Università e Ricerca Scientifica e Tecnologica (MURST 60%).

### REFERENCES

Alanko A, Heinonen E, Scheinin T, Tolppanen EM and Vihko R (1985) Significance of estrogen and progesterone receptors, disease-free interval, and site of first metastasis on survival of breast cancer patients. *Cancer* **56**: 1696–1700

Allegra JC, Lippman ME, Thompson EB, Simon R, Barlock A, Green L, Huff KK, Do HM and Aitken SC (1979) Distribution, frequency, and quantitative analysis of estrogen, progesterone, androgen, and glucocorticoid receptors in human breast cancer. *Cancer Res* **39**: 1447–1454

Anelli TF, Anelli A, Tran KN, Lebwohl DE and Borgen PI (1994) Tamoxifen administration is associated with a high rate of treatment-limiting symtoms in male breast cancer patients. *Cancer* **74**: 74–77

Bertuzzi A, Daidone MG, Di Fronzo G and Silvestrini R (1981) Relationship among estrogen receptors, proliferative activity and menopausal status in breast cancer. *Breast Cancer Res Treat* **1**: 253–262

Bezwoda WR, Hesdorffer C, Dansey R, de Moor N, Derman DP, Browde S and Lange M (1987) Breast cancer in men. Clinical features, hormone receptor staus, and response to therapy. *Cancer* **60**: 1337–1340

Birrell SN, Roder DM, Horsfall DJ, Bentel JM and Tilley WD (1995) Medroxyprogesterone acetate therapy in advanced breast cancer: the predictive value of androgen receptor expression. *J Clin Oncol* **13**: 1572–1577

Bruce DM, Heys SD, Payne S, Miller ID and Eremin O (1996) Male breast cancer: clinico-pathological features, immunocytochemical characteristics and prognosis. *Eur J Surg Oncol* **22**: 42–46

Bryan RM, Mercer RJ, Bennett RC, Rennie GC, Lie TH and Morgan FJ (1984) Androgen receptors in breast cancer. *Cancer* **54**: 2436–2440

Calandra RS, Charreau EH, Royer de Giaroli M and Baldi A (1984) Incidence of estrogen, progesterone and prolactin receptors in human breast cancer. *Prog Clin Biol Res* **142**: 97–108

Cox DR (1972) Regression models and life tables (with discussion). *J R Stat (Series B)* **34**: 187–220

Cutuli B, Lacroze M, Dilhuydy JM, Velten M, De Lafontan B, Marchal C, Resbeut M, Graic Y, Campana F, Moncho-Bernier V, De Gislain G, Tortochaux J, Cuillere JC, Reme-Saumon M, N'Guyen TD, Lesaunier F, Le Simple T, Gamelin E, Henry M and Berlie J (1995) Male breast cancer: results of the treatments and prognostic factors in 397 cases. *Eur J Cancer* **31A**: 1960–1964

Dawson PJ, Paine TM and Wolman SR (1992) Immunocytochemical characterization of male breast cancer. *Modern Pathol* **5**: 621–625

Dixon WJ, Brown MG, Engelman L, Hill MA and Jennrich RI (1990) *BMPD Statistical Software Manual*. University of California Press: Berkeley

Elston CW and Ellis IO (1991) Pathological prognostic factors in breast cancer. I. The value of histological grade in breast cancer: experience from a large study with long-term follow-up. *Histopathology* **19**: 403–410

Everson RB, Lippman ME and Thompson EB (1980) Clinical correlations of steroid receptors and male breast cancer. *Cancer Res* **40**: 991–997

Fox SB, Rogers S, Day CA and Underwood JCE (1992) Oestrogen receptor and epidermal growth factor receptor expression in male breast carcinoma. *J Pathol* **166**: 13–18

Friedman MA, Hoffman PG, Dandolos EM, Lagios MD, Johnston WH and Siiteri PK (1981) Estrogen receptors in male breast cancer: clinical and pathologic correlations. *Cancer* **47**: 134–137

Gerdes J, Pickartz H, Brotherton J, Hammerstein J, Weitzel H and Stein H (1987) Growth fractions and estrogen receptors in human breast cancers as determined in situ with monoclonal antibodies. *Am J Pathol* **129**: 486–492

Guinee VF, Olsson H, Moller T, Shallenberger RC, van den Blink JW, Peter Z, Durand M, Dische S, Cleton FJ, Zewuster R, Fang Cui M, Lane W and Richter R (1993) The prognosis of breast cancer in males. *Cancer* **71**: 154–161

Hähnel R (1985) Progesterone receptor assay in the management of breast and other cancers. *Rev Endocr Relat Cancer* **20**: 5–11

Hecht JR, Winchester DJ (1994) Male breast cancer. *Am J Clin Pathol* **102** (suppl. 1): 25–30

Hermanek P and Sobin LH (1992) *TNM Classification of Malignant Tumours,* 4th edn. Springer-Verlag: New York

Hiort O, Kwan PWL and DeLellis RA (1988) Immunohistochemistry of estrogen receptor protein in paraffin sections. *Am J Clin Pathol* **90**: 559–563

Isola JJ (1993) Immunohistochemical demonstration of androgen receptor in breast cancer and its relationship to other prognostic factors. *J Pathol* **170**: 31–35

Jacobs TW, Prioleau JE, Stillman IE and Schnitt SJ (1996) Loss of tumor marker-immunostaining intensity on stored paraffin slides of breast cancer. *J Natl Cancer Inst* **88**: 1054–1059

Joshi MG, Lee AK, Loda M, Camus MG, Pedersen C, Heatley GJ and Hughes KS (1996) Male breast carcinoma: an evaluation of prognostic factors contributing to a poorer outcome. *Cancer* **77**: 490–498

Kaplan EL and Meier P (1958) Non parametric estimation for incomplete observations. *J Am Stat Assoc* **53**: 457–481

Kuenen-Boumeester V, Van der Kwast TH, Claassen CC, Look MP, Liem GS, Klijn JG and Henzen-Logmans SC (1996) The clinical significance of androgen receptors in breast cancer and their relation to histological and cell biological parameters. *Eur J Cancer* **32A**: 1560–1565

Langer M, Kubista E, Schemper M and Spona J (1990) Androgen receptors, serum androgen levels and survival of breast cancer patients. *Arch Gynecol Obst* **247**: 203–209

Lea OA, Kvinnsland S and Thorsen T (1989) Improved measurement of androgen receptors in human breast cancer. *Cancer Res* **49**: 7162–7167

McGuire WL and Clark GM (1985) The role of progesterone receptors in breast cancer. *Semin Oncol* **12** (suppl.): 12–16

Mercer RJ, Bryan RM and Bennett RC (1984) Hormone receptors in male breast cancer. *Aust NZ J Surg* **54**: 215–218

Miller WR, Telford J, Dixon JM and Hawkins RA (1985) Androgen receptor activity in human breast cancer and its relationship with oestrogen and progestogen receptor activity. *Eur J Cancer Clin Oncol* **21**: 539–542

Millis RR (1980) Correlation of hormone receptors with pathological features in human breast cancer. *Cancer* **46**: 2869–2871

Nomura Y, Yamagata J, Takenaka K and Tashiro H (1980) Steroid hormone receptors and clinical usefulness in human breast cancer. *Cancer* **46** (suppl.): 2880–2883

Pacheco MM, Oshima CF, Lopes MP, Widman A, Franco EL and Brentani MM (1986) Steroid hormone receptors in male breast diseases. *Anticancer Res* **6**: 1013–1017

Pertschuk LP, Kim DS, Nayer K, Feldman JG, Eisenberg KB, Carter AC, Rong ZT, Thelmo WL, Fleisher J and Greene GL (1990) Immunocytochemical estrogen and progestin receptor assays in breast cancer with monoclonal antibodies. *Cancer* **66**: 1663–1670

Pertschuk LP, Feldman JG, Kim YD, Braithwaite L, Schneider F, Braverman AS and Axiotis C (1996) Estrogen receptor immunocytochemistry in paraffin embedded tissues with ER1D5 predicts breast cancer endocrine response more accurately than H222Spγ in frozen sections or cytosol-based ligand-binding assays. *Cancer* **77**: 2514–2519

Pich A, Margaria E and Chiusa L (1994) Proliferative activity is a significant prognostic factor in male breast carcinoma. *Am J Pathol* **145**: 481–489

Pich A, Margaria E, Chiusa L, Ponti R and Geuna M (1996) DNA ploidy and p53 expression correlate with survival and cell proliferative activity in male breast carcinoma. *Hum Pathol* **27**: 676–682

Poulin R, Baker D and Labrie F (1988) Androgens inhibit basal and oestrogen-induced cell proliferation in the ZR-75-1 human breast cancer cell line. *Breast Cancer Res Treat* **12**: 213–225

Prioleau JE and Schnitt SJ (1995) p53 antigen loss in stored paraffin slides (letter). *N Engl J Med* **332**: 1521–1522

Ribeiro G (1985) Male breast cancer: review of 301 cases from Christie Hospital and Holt Radium Institute, Manchester. *Br J Cancer* **51**: 115–119

Ribeiro G and Swindell R (1992) Adjuvant tamoxifen for male breast cancer (MBC). *Br J Cancer* **65**: 252–254

Rogers S, Day CA and Fox SB (1993) Expression of cathepsin D and estrogen receptor in male breast carcinoma. *Hum Pathol* **24**: 148–151

Salvadori B, Saccozzi R, Manzari A, Andreola S, Conti RA, Cusumano F and Grassi M (1994) Prognosis of breast cancer in males. An analysis of 170 cases. *Eur J Cancer* **30**: 930–935

Sasano H, Kimura M, Shizawa S, Kimura N and Nagura H (1996) Aromatase and steroid receptors in gynecomastia and male breast carcinoma: an immunohistochemical study. *J Clin Endocrinol Metab* **81**: 3063–3067

Scarff RW and Torloni H (1968) *Histological Typing of Breast Tumours*. International histological classification of tumours. No. 2. World Health Organization: Geneva

Soreide JA, Lea OA, Varhaug JE, Skarstein A and Kvinnsland S (1992) Androgen receptors in operable breast cancer: relation to other steroid hormone receptors, correlations to prognostic factors and predictive value for effect of adjuvant tamoxifen treatment. *Eur J Surg Oncol* **18**: 112–118

Teller MN, Stock CC, Stohr G, Merker PC, Kaufman RJ, Escher GC and Bowie M (1966) Biologic characteristics and chemotherapy of 7,12-dimethylbenz-(α)anthracene-induced tumors in rats. *Cancer Res* **26**: 245–252

Walker KJ, Bouzubar N, Robertson J, Ellis IO, Elston CW, Blamey RW, Wilson DW, Griffiths K and Nicholson RI (1988) Immunocytochemical localization of estrogen receptor in human breast tissue. *Cancer Res* **48**: 6517–6522

Weber-Chappuis K, Bieri-Burger S and Hurlimann J (1996) Comparison of prognostic markers detected by immunohistochemistry in male and female breast carcinomas. *Eur J Cancer* **32**: 1686–1692

Wilbur DC, Willis J, Mooney RA, Fallon MA, Moynes R and di Sant'Agnese A (1992) Estrogen and progesterone receptor detection in archival formalin-fixed, paraffin embedded tissue from breast carcinoma: a comparison of immunohistochemistry with the dextran-coated charcoal assay. *Modern Pathol* **5**: 79–84

Williams Jr WL, Powers M and Wagman LD (1996) Cancer of the male breast: a review. *J Natl Med Assoc* **88**: 439–443

Willsher PC, Leach IH, Ellis IO, Bell JA, Elston CW, Bourke JB, Blamey RW and Robertson JF (1997) Male breast cancer: pathological and immunohistochemical features. *Anticancer Res* **17**: 2335–2338

*© Cancer Research Campaign 1999*
